# What is schizophrenia: A neurodevelopmental or neurodegenerative disorder or a combination of both? A critical analysis

**DOI:** 10.4103/0019-5545.58891

**Published:** 2010

**Authors:** Swapnil Gupta, Parmanand Kulhara

**Affiliations:** Department of Psychiatry, Postgraduate Institute of Medical Education and Research, Chandigarh - 160 012, India

**Keywords:** Schizophrenia, neurodevelopmental, neurodegeneration

## Abstract

The etiology of schizophrenia has been the focus of intensive research for a long time. Perspectives have changed drastically with the development of new investigative techniques. Clinical observations made by Kraepelin, Clouston, Bender, and Watt are now being complemented by neuroimaging and genetic studies to prove the neurodevelopmental hypothesis. At the same time, neuropathological and longitudinal studies of schizophrenia often support a neurodegenerative hypothesis. To provide a theoretical basis to the available evidence, another hypothesis called the progressive neurodevelopmental model has also emerged. This review presents some key evidence supporting each of these theories followed by a critical analysis of each.

## INTRODUCTION

Schizophrenia is a chronic and disabling mental illness affecting millions of people worldwide. The study of the etiology of schizophrenia is ongoing although perspectives have changed. Various factors ranging from psychodynamic to autoimmune to genetic have been reported to be invoked in the causation of this disorder.

The proposition that schizophrenia may have its roots in early brain development dates back to Kraepelin and Bleuler, both having noted neurological and behavioral abnormalities in the childhood histories of their adult patients. Several others like Bender[1] and Watt[2] described similar abnormalities, but the theory that schizophrenia might be a developmental disorder was first proposed by Thomas Clouston[3] who called it a “developmental insanity”. Advancing investigative technology spawned enthusiastic research in the neurobiology of schizophrenia. As scientists uncovered more facts about normal neural development and neurogenesis, the neural abnormalities found in schizophrenia were thought to be a result of aberrant neurodevelopment. As this course was pursued more actively, more and more evidence supporting a neurodevelopmental theory of schizophrenia emerged. Evidence accumulated from various types of studies, including those of obstetric complications, facial dysmorphogenesis, genetic neuroimaging, and neuropathological studies. This theory, however, left several vital questions unanswered. For example, how could a purely neurodevelopmental disorder manifest for the first time in an adolescent or an adult?

The neurodegenerative hypothesis has its beginnings in the descriptions of schizophrenia given by Kraepelin who popularized the term “dementia praecox,” where inherent to its definition was the assertion of a chronic downhill course that is typical of neurodegenerative disorders. Studies of various types including neuroimaging, cognitive functions, and postmortem brain histopathology have been done to find support for this hypothesis.

Thorough analyses of both these theories as well as the redefinition of the concepts of neurodevelopment and neurodegeneration have led to a third possibility. A unifying hypothesis has been proposed that conceptualizes schizophrenia as a progressive neurodevelopmental disorder. The term may at first glance seem contradictory but has been made acceptable by the redefinition of the boundaries of neurodevelopment and neurodegeneration.

The following review attempts to re-examine the strengths and weaknesses of both these theories. A head-to-head comparison of the two theories is fallacious for two reasons: (i) No researcher has ever claimed that schizophrenia is entirely neurodevelopmental or solely neurodegenerative, (ii) these theories are complementary rather than exclusive, because they can individually explain certain phenomena observed in the onset and course of schizophrenia, and put together, they can explain the onset as well as the course and outcome.

### Normal neurodevelopment

It must be remembered that development is a process that occurs in the normal human brain as a function of age. Hence, any pathology or deviance is closely entwined with the age-associated stage of development or degeneration of the brain. There are some important facts about normal neurodevelopment: (i) Different brain regions are generated at different times during development. Hence, the timing of the insult to the growing fetus in intrauterine life is a major determinant of the subsequent anomaly observed. (ii) Minor abnormalities in early events can produce large differences in subsequent stages. (iii) Specific molecular signals play specific roles at various stages during neurodevelopment. For example, noggin and follistatin cause induction of the central nervous system; brain-derived neurotrophic factor (BDNF) and Insulin-like growth factor (IGF) are the major signals for proliferation. Proteins such as reelin and astrotactin cause migration of the growing neurons to the appropriate positions in the brain. Signals from interneurons assume importance in the process of pruning that occurs in adolescence. All of these proteins are controlled by specific genes.

### Neurodevelopmental disorders

The term, “neurodevelpmental disorder” implies that the brain is not formed normally from the beginning. Abnormal regulation of fundamental neurodevelopmental processes may occur, or there may be disruption by insult that may take various forms. Autism and attention deficit hyperactivity disorder have been classically described as neurodevelopmental disorders.

### Is schizophrenia a neurodevelopmental disorder?

Thomas Clouston had called schizophrenia the last cortical developmental disease. Southard had discovered some neuropathological abnormalities that he interpreted as being suggestive of neurodevelopmental defects. Schizophrenia had also been called as a developmental encephalopathy.[1] Several neurological and social developmental abnormalities were also found in the children who went on to develop schizophrenia.[4] Such initial observations were followed by more robust evidence that can be grouped as follows:

1. Obstetric complications
2. Clinical signs of aberrant neurodevelopment in the form of soft neurological signs and social and intellectual deficits
3. Neuroimaging of first-episode psychosis and childhood onset schizophrenia
4. Postmortem neuropathological studies
5. Genes
6. Developmental proteins

#### Obstetric complications

An association of schizophrenia with complications of pregnancy and delivery was proposed by Rosanoff as early as 1934. This was followed by several register-based cohort studies that showed an association between the two.[5–7] Several prospective cohort studies also showed increased risk of developing schizophrenia when the rate of obstetric complications was higher.[8,9] A metanalysis by Geddes *et al*.[10] showed that the pooled odds ratio of 23 studies was 2. However, the authors cautioned against a publication bias for positive studies. Another metanalysis by Cannon *et al*.[8] documented that the pooled effect sizes were < 2. Various complications implicated in these studies were bleeding during pregnancy, abruption, hypertension and the use of diuretics, preeclampsia, and polyhydraminos.

Some studies have also shown an association between maternal influenza infections and the development of schizophrenia in the offspring.[11–13]

However, almost an equal number of studies have refuted these findings.[14–16] A study of maternal malnutrition conducted during a famine in the Netherlands also found an association of maternal malnutrition with schizophrenia.[17]

#### Minor physical anomalies

High palate was observed in many schizophrenia patients which was indicative of abnormal development of the branchial arches.[18] A study which attempted to segregate patients of schizophrenia from those without the illness based on facial features, showed that a combination of 12 variables related to cranial morphology predicted correct classification of 90% of schizophrenia patients. The variables included high-arched palate, low-set ears, altered hairline, and palpebral fissure anomalies.[19] Altered dermatoglyphic patterns were seen in schizophrenia patients.[20,21]

#### Premorbid neurological and behavioral abnormalities

A large number of follow-up studies identified that certain abnormalities in intellectual and social functioning were more common in children who went on to develop schizophrenia in later life. Some of the major studies and their findings are shown in [Table 1].[22]

**Table 1:** Studies demonstrating premorbid deficits in social, neurological, and intellectual functioning in schizophrenia[22]

Study	Sample and F/U	Findings
British birth cohort 1946 (Jones, 1994)*	5000/40 yrs	Delayed milestones, speech problems, poor social competence
David *et al*., (1997)*	Swedish army conscripts 50000/12 yr	Low IQ at 18 yrs
Davidson (1999)*	Israeli army conscripts 10000	IQ, social functioning, organizational ability
N. Finland cohort 1966 (Isohanni, 2003)*	12000 babies/31 yrs	Delayed milestones

* Cited by Murray and Bramon[22]

#### Neuroimaging studies

Numerous neuroimaging studies supporting neurodevelopmental hypothesis are available. However, studies of first-episode psychosis can be considered to be more suggestive of a neurodevelopmental rather than a neurodegenerative process because these studies do not reflect the effect of the disease on the brain after it has begun. Several studies also eliminate the effect of substance abuse and medication. The MRI studies in first-episode schizophrenia mentioned in a comprehensive review on this subject by Shenton *et al*.[23] are shown in [Table 2].

**Table 2:** Cross-sectional MRI studies of schizophrenia[23]

Finding	Positive studies	Negative studies
Hippocampus	9	2
Frontal lobe	5	4
Basal ganglia	6	3
Cavum septum pellucidi	3	None

The fact that differences in size were observed between patients of schizophrenia and age-matched normal controls in various structures such as the hippocampus and the basal ganglia suggest that the development of these structures may have been abnormal. More severe developmental anomalies were seen in childhood-onset schizophrenia than in the adult-onset type. There were more cavum septum pellucidi anomalies than in normal controls[24] but lesser cerebral volumes and no hippocampal asymmetry.[25]

#### Postmortem neuropathological studies

Ectopic cortical neurons and abnormal cortical cytoarchitecture were found in the prefrontal lobe in one study, which suggested abnormalities of neuronal migration.[26] This finding could not be replicated by another study.[27] Abnormal sulco-gyral patterns were observed in the entorhinal cortex in one study,[28] but this was not subsequently replicated by four other studies.[29–32] This inconsistency was defended by some researchers who pointed out that the entorhinal cortex showed marked morphological heterogeneity even in normal individuals. They[29,30] also reported that studies of the neuropathology of schizophrenia presented special technical challenges because the exact regions of interest were relatively unknown and the preservation and staining techniques used for such studies were still considered deficient. Thus, the lack of consistent findings in this area should not be considered as strong evidence against the neurodevelopmental hypothesis.

#### Schizophrenia susceptibility genes related to neurodevelopment

As mentioned earlier, all the molecular signals involved in neurodevelopment are controlled by specific genes. Some of the genes that have been identified as candidate genes in schizophrenia have also been clearly shown to be linked to the process of neurodevelopment. The genes and their functions have been listed in [Table 3].[33]

**Table 3:** Genes associated with schizophrenia[33]

Gene	Function	Study
DISC-1	Neuronal migration	Kamiya *et al*., 2005
	Synaptic organization	Kirkpatrick *et al*., 2006
NRG-1 (neuregulin-1)	Neuronal migration	Anton *et al*., 1997
	Myelination	Traveggia *et al*., 2005
DTNBP-1 (dysbindin-1)	Synaptic plasticity	Talbot *et al*., 2004

#### Developmental proteins

Abnormalities of proteins that are specifically involved in the development of the human brain have been seen in patients with schizophrenia. Levels of reelin, a protein involved in cell migration and plasticity, are found to be reduced.[34] Levels of a polysialated neural cell adhesion molecule (PSA-N-CAM), which is involved in axon and dendrite formation, were found to be increased in one study[35] but decreased in another.[36] Other proteins that are implicated are Brain-derived Neurotrophic Factor (BDNF), Glial-derived Neurotrophic Factor (GDNF), Epithelial Growth Factor (EGF), and Basic Fibroblast Growth Factor (bFGF).

### The late neurodevelopmental model

During normal adolescence, changes are observed that are indicative of changes in the structure and function of the brain. There is a decrease in delta sleep in the sleep EEG,[37] a decrease in membrane synthesis,[38] decreased cortical gray matter volume,[39] and decreased prefrontal metabolism.[40] In schizophrenia, there are more pronounced decrements in the same parameters.[38,41–43] This supports the possibility of an exaggeration of the normal process of synaptic pruning that occurs as a normal process during adolescence. Neural network modelling studies of schizophrenia also support the hypothesis of exaggerated pruning.[44] It has also been suggested that there is a reduction in the synapse-rich neuropil and a consequent increase in cortical neuron density. This is called the reduced neuropil hypothesis.[45]

### Normal ageing and abnormal neurodegeneration

Identifying a boundary between the two processes is difficult. The changes that occur are mainly in the following domains:

1. Total neuronal volume
2. Neuronal structure
3. Neurotransmitters/receptors/neurotrophins/second messenger system
4. Metabolic changes—oxidative stress
5. Vascular changes

### Neurodegenerative diseases

These are chronic progressive disorders of the nervous system that affect neurological and behavioural function and involve biochemical changes leading to distinct histopathologic and clinical syndromes.[46] Abnormal proteins resistant to cellular degradation mechanisms accumulate within the cells. The pattern of neuronal loss is selective in the sense that one group gets affected, whereas others remain intact. Often, there is no clear inciting event for the disease. The diseases classically described as neurodegenerative are Alzheimer's disease, Huntington's disease, and Parkinson's disease.

### Is schizophrenia a neurodegenerative disorder?

It was earlier believed that the course of schizophrenia was always progressive and improvement was not possible. Kraepelin had called schizophrenia *“Dementia praecox,”* implying a progressive deterioration from which recovery was not possible. On examining the clinical course of the disorder, we see that some patients have a deteriorating rather than a static course; a subgroup of patients has a chronic course with multiple exacerbations. The likelihood of deterioration correlates with the number of periods and the duration of positive symptoms.[47]

Early treatment with antipsychotic medications may arrest this progression. A longer duration of untreated psychosis predicts a poorer outcome, suggesting that there are possible adverse neurotoxic effects of untreated psychosis. Long DUP predicted worse response to medications, higher relapse risk, and mixed association with other outcome measures.[48] This indicates that a pathological process is occurring in the brain, against which drugs played a protective role. It is also seen that patients appear to take longer to recover and show less complete recovery over successive episodes of this illness.[49]

Studies of cognitive functions were equivocal and did not clearly support either hypothesis. Studies showed that baseline cognitive functions in patients were one SD below the values for normal controls. There was little evidence for deterioration of cognitive functions over five years after onset, except language functions. [Table 4] shows some of the longitudinal studies of cognitive function done in patients of schizophrenia.

**Table 4:** Longitudinal studies of cognitive function in schizophrenia

Study	Sample and F/U	Findings
Heaton *et al*. (2001)[50]	142 schizophrenia patients, 206 normal controls followed up for 3 yrs	Improvement after treatment then stable
Hoff *et al*. (2005)[51]	42 schizophrenia patients, 16 normal controls, followed up for 10 yr	Improvement on treatment then stable
Gold *et al*. (1999)[52]	54 schizophrenia patients	Functions improved after treatment except language

#### Neuroimaging studies

Longitudinal neuroimaging studies using techniques like MRI have been done. Longitudinal studies show changes that occur in the brain after the illness has begun, thereby representing the effect of the illness on the brain. [Table 5] shows some of these studies along with the findings.

**Table 5:** Longitudinal neuroimaging studies in schizophrenia[23]

Study	Sample/F/U	Finding
Keshavan *et al*. (1998)*	17 schizophrenia patients/17 normal controls, 1 yr	Rate of change of temporal lobe size more in SZ
Rapoport *et al*. (1997)*	16 childhood onset schizophrenia patients/24 normal controls, 2 yrs	Rate of change of frontal lobe size and thalamic area more in COSZ
Rapoport *et al*. (1998)*	15 childhood onset schizophrenia/34 normal controls, 4 yrs	Rate of change of grey matter (frontal, temporal and parietal) more in COSZ

* cited by Shenton *et al*.[23]

#### Postmortem studies

Gliosis is a cardinal feature of neurodegeneration. There is strong evidence for its absence in schizophrenia. It has been suggested that neurodegeneration in schizophrenia is caused by glutamatoxicity which causes a graded apoptosis and hence, is not followed by gliosis. Doubts have also been raised regarding the sensitivity of the histopathological methods used to detect gliosis.[53]

#### Neurochemical changes

Dopamine-mediated neurochemical sensitization is the earliest neurochemical theory of schizophrenia.[54] Of late, the involvement of other neurotransmitters is being highlighted. Excess glutamate leading to apoptosis, followed by calcium release and oxidative damage has been seen. NMDA receptor hypofunction is a theory proposed on the basis of the observation that antagonism of NMDA receptors with drugs like ketamine causes psychotic symptoms.[55] GABA interneuron-mediated inhibition of pyramidal neurons has also been seen to be reduced.[56]

#### Metabolic changes

Levels of ntioxidant enzymes such as superoxide dismutase (SOD), reduced glutathione (GSH), and catalase, and nonenzymatic antioxidants such as ascorbate, albumin, and selenium have been found to be reduced. Positive symptoms of schizophrenia correlate inversely with levels of superoxide dismutase whereas negative symptoms correlate inversely with levels of reduced glutathione. Haloperidol treatment led to an increase in SOD activity.[57] Oxidative stress is a prominent finding in any type of degenerative process and hence, these changes support a degenerative hypothesis.

### Schizophrenia as a progressive developmental disease

This theory developed after a large amount of data had been gathered, and emerged as an attempt to synthesize conflicting findings. A progressive developmental hypothesis reconciles the contradictory imaging and neuropathological data.[58] Schizophrenia is better viewed as a lifetime disorder of development, plasticity, and ageing with windows of vulnerability at all three stages of life [Table 6].[48]

**Table 6:** Clinical and pathological stages of schizophrenia[49]

Clinical stage	Pathological stage
Premorbid	Neurodevelopmental
Prodromal	Neuronal maturational events
Onset/deteriorative	Endogenous neurochemical sensitization
Chronic/residual	Neuroprogression—toxicity

#### Dopaminergic hypothesis

Dopamine is the most extensively investigated neurotransmitter in schizophrenia. The dopaminergic hypothesis came about from the observation that drugs that antagonized dopamine were found to be effective in the treatment of schizophrenia. This theory dominated the scene for nearly fifteen years. The evidence for the role of dopamine in the pathogenesis of schizophrenia comes from the fact that there are abnormalities in genes involved in dopamine synthesis, receptors and transporters, functional neuroimaging studies (SPECT and PET), and the efficacy of antidopaminergic agents in treating schizophrenia.

Dopamine has also been implicated in mediating aberrations in i) developmental processes such as neuronal proliferation and migration as well as pruning, and ii) degenerative processes such as oxidative stress and excitotoxicity. In adolescence, the onset of psychosis may be related to an excessive elimination of synapses and secondarily, phasic dopaminergic overactivity. This hypothesis is consistent with central characteristics of schizophrenia such as premorbid manifestations, adolescent onset, functional decline early in the illness, cognitive impairments, the role of dopamine, and the role of genes and environment in pathophysiology. Dopamine neurons to the prefrontal cortex from the striatum are under tonic excitatory control of NMDA and non-NMDA glutamatergic neurons.[59] Defective functioning of the corticostriatal glutamatergic systems could, therefore, lead to reduced tonic dopamine release, and in turn, increase phasic stress-induced dopamine release. Such dysregulation of the tonic-phasic DA system has been proposed to account for the positive and negative symptoms of schizophrenia that emerge during adolescence.[60] If untreated, persistent dopaminergic and consequent ‘phasic’ glutamatergic excess could lead to further excitotoxic brain damage by increasing oxidative stress.

#### Glutamate: Is it the bridge?

It has been postulated that glutamate could be the link between neurodevelopment and neurodegeneration. It plays a role in several stages of neurodevelopment (neuronal migration, survival, and plasticity). In adolescence, it is involved in plasticity and pruning, and in old age, it is implicated in neurodegeneration through excitotoxicity. Thus, a “three hit hypothesis” has been proposed for the role of glutamate in schizophrenia.[49] More evidence for the role of glutamate comes from the fact that drugs modulating glutamate receptors and trophic hormones modulate synaptic pruning processes. The effects of second generation antipsychotic medications are linked to their ability to modulate glutamatergic neurotransmission also. Molecules that enhance glutamatergic transmission, *e.g*., glycine, serine, and cycloserine are being explored for the treatment of negative and cognitive symptoms.

### Critical analysis

#### Why do we need to know this?

If schizophrenia is a purely developmental disorder, our focus will be limited to understanding its etiology and refining preventive strategies. If a neurodegenerative element is present, then we will focus on prevention, early intervention, and treatment strategies. The label of developmental disorder also conveys a sense of therapeutic nihilism which may be detrimental. The presence of neurodevelopmental anomalies does not rule out neurodegeneration as a significant presence in schizophrenia and *vice versa*. Direct comparisons of the theories are not possible but the strengths and weaknesses of each theory can be examined.

#### Neurodevelopmental hypothesis

There is evidence of aberrations in the same process from various perspectives (etiological, genetic, histopathological, neuroanatomical, and clinical). This hypothesis claims that schizophrenia is a disorder of brain development. Hence, by definition, the disease should be early-onset *not* late-onset, untreatable *not* treatable, and static *not* progressive.

#### Neurodevelopmental hypothesis—Onset of illness

Arguments that go against a neurodevelopmental hypothesis of schizophrenia are many: i) The typical age of onset for the illness is adolescence or early adulthood, ii) there is adequate evidence for late-onset cases, iii) the illness causes a marked deterioration from premorbid levels and not merely an inability to cope with peers. The hypothesis is able to address these issues by using three convincing arguments: i) Premorbid abnormalities in the nervous system and behavior do exist in schizophrenia. ii) The late neurodevelopmental hypothesis explains why the disease manifests in adolescence and early adulthood. iii) Early damage to neurons can lead to manifest pathology on interaction with normal maturational events.

#### Neurodevelopmental hypothesis—Treatment?

Certain aspects of the “treatability” of schizophrenia go against the neurodevelopmental hypothesis. There is clear evidence that antipsychotic agents work. It has also been seen that early institution of antipsychotics leads to a less malignant course of the illness. There is also evidence to show that a sizeable proportion of patients recover completely.

### Neurodegenerative hypothesis

This hypothesis proposes that schizophrenia is a disorder caused due to the degeneration of the brain. By definition, the disease should have characteristic histopathological features and progression.

#### Neurodegenerative hypothesis—histopathology

Absence of gliosis and of any other histological evidence of degeneration such as inclusion bodies, is the strongest argument against neurodegeneration. This has been explained by “pathological neuronal apoptosis” instead of necrosis; apoptosis will not cause gliosis. A genetic defect in *bcl-2* has been seen in schizophrenia which supports pathological apoptosis. The sensitivity of methods to detect gliosis has been questioned. Subcellular biochemical evidence of degeneration in the form of oxidative damage has been seen in schizophrenia.

#### Neurodegenerative hypothesis—progression

Longitudinal neuroimaging studies have been equivocal and generally, no progressive deterioration has been seen in cognitive functions. However, subgroups with clinical, cognitive, and neuroimaging evidence of progression are present.

Thus, evidence from histopathology is mostly against neurodegeneration but clinical and biochemical evidence of degeneration is undeniably present in certain groups of patients with schizophrenia.

### Progressive neurodevelopmental disorder

This term and concept are new and other disorders that may be classified in the same group are few. It may be said that it has been specially created to accommodate findings in the study of schizophrenia. The theoretical bases of this hypothesis are evolving and its biggest strength is that the theory emerges from research findings and not *vice versa*. It may be said that the disorder is unique and hence, requires a unique biological explanation.

## CONCLUSIONS

Schizophrenia is a complex and unique disorder and probably cannot be explained by a single process of development or degeneration. Research evidence exists for degeneration as well as development, although at present, evidence for the latter appears to be stronger. There is considerable heterogeneity in clinical findings; there may be different subgroups with different contributions of various processes towards disease manifestation. Of late, there are theoretical proposals such as the glutamatergic hypothesis that bridge the gap between development and neurodegeneration. Evidence for this proposition at present is minimal. Finally, it should be remembered that viewing schizophrenia as having both components of development and degeneration is therapeutically more optimistic.
